# Normal Saline Versus Balanced Crystalloids in Renal Transplant Surgery: A Double-Blind Randomized Controlled Study

**DOI:** 10.7759/cureus.18247

**Published:** 2021-09-24

**Authors:** Vikas Saini, Tanvir Samra, Naveen Naik B, Venkata Ganesh, Kashish Garg, Sameer Sethi, Deepesh B Kanwar, Sarbpreet Singh, Neha Rawat, Deepkanth Gorla

**Affiliations:** 1 Anaesthesia and Intensive Care, Postgraduate Institute of Medical Education and Research, Chandigarh, IND; 2 Renal Transplant Surgery, Postgraduate Institute of Medical Education and Research, Chandigarh, IND

**Keywords:** potassium, normal saline, acidosis, hyperchloremia, perioperative, renal transplant, plasmalyte, balanced salt solutions

## Abstract

Background

The importance of optimal acid-base balance during renal transplant surgeries cannot be stressed enough. Optimal preload and electrolyte balance is important in maintaining this. There has been a debate on the choice of perioperative crystalloids in renal transplant surgeries over the past decades. Normal saline (0.9% saline) is more likely to cause hyperchloremic acidosis when compared to balanced salt solutions (BSS) with low chloride content whereas BSS may cause hyperkalemia. We aim to compare the safety and efficacy of normal saline (NS), Ringer’s lactate (RL) and Plasmalyte (PL) on acid-base balance and electrolytes during living donor kidney transplantation.

Materials and methods

Patients were randomized to NS group (n = 60), RL group (n = 60) and Plasmalyte group (n = 60). Arterial blood samples were collected for acid-base analysis after induction of anaesthesia (T0), prior to clamping the iliac vein (T1), 10 minutes after reperfusion of the donated kidney (T2) and at the end of surgery (T3). In addition, serum creatinine and 24-hour urine output were recorded on postoperative days one, two and seven.

Results

There was a statistically significant difference (p < 0.001) in the pH at the end of surgery between the three groups with the NS group being more acidotic (pH 7.29 ± 0.06, 95% CI 7.27-7.32), although this was not clinically relevant. This was explainable by the parallel increase in chloride in the NS group. Early postoperative graft functions in terms of serum creatinine, urine output and graft failure requiring dialysis were not significantly different between the groups.

Conclusion

Balanced salt solutions such as Plasmalyte and Ringer’s lactate are associated with better pH and chloride levels compared to normal saline when used intraoperatively in renal transplant patients. This difference, however, does not appear to have any bearing on graft function. Plasmalyte seems to maintain a better acid-base and electrolyte balance, especially during the postreperfusion period.

## Introduction

Intravenous fluid therapy is a ubiquitous, modifiable component of perioperative living donor kidney transplantation. Renal transplant surgery patients receive large volumes of intravenous fluids. On average, these patients are given around 3,000 mL of crystalloid intravenous fluids intraoperatively and around 2,500 mL in the first 24 hours after surgery. There is marked heterogeneity in clinical practice and no consensus on the appropriate choice and volume of fluid to be given in renal transplant surgical patients. For optimal graft function in the post-transplant period, adequate intravascular volume, electrolyte and homeostasis need to be maintained. To prevent renal insufficiency and electrolyte disturbances like hyperkalemia and hyperchloremia, a good understanding and management of fluid therapy are required. Normal saline has traditionally been the fluid of choice in the perioperative period of kidney transplantation. Normal saline infusion has been known to cause hyperchloremia [1]. This hyperchloremia has the potential to cause renal hypoperfusion and precipitate acute kidney injury and associated 2.05 odds of higher 30-day mortality [2,3]. Balanced salt solutions do not affect serum potassium levels any more than normal saline while maintaining a better acid-base balance in these patients.

Limited research and the absence of robust evidence on the appropriate type of intravenous fluid have resulted in an ongoing debate on the appropriate use of intravenous fluids for living donor kidney transplantation. The purpose of this study is to compare the effects of normal saline, Ringer's lactate and Plasmalyte on acid-base balance and electrolytes during living donor kidney transplantation.

## Materials and methods

This study was commenced after approval from the Institute Ethics Committee (IEC/2016/937) and is registered in the Clinical Trials Registry (NCT03115060). Written informed consent was obtained from every patient included in the study. This prospective, randomized, double-blind study was conducted between April 2015 and December 2018 at the renal transplant centre in cooperation with the department of renal transplant surgery at a tertiary care centre in Chandigarh that caters to over 200 renal transplant recipients yearly.

Inclusion criteria and randomization

Patients aged 20-60 years with the American Society of Anesthesiologists (ASA) physical status III and IV and scheduled for living-related kidney transplantation were included. Participants were excluded if they had (i) cardiovascular, respiratory, metabolic, hematologic, hepatic, or central nervous system diseases; (ii) pre-operative serum potassium level of more than 5.5 mEq/L; and (iii) refusal of study participation.

Computer-based randomization (block randomization) was performed and allocation into one of three treatment groups was done using sealed envelopes. The patient and the investigator performing the analysis were blinded to the allocation. The perioperative staff was, however, not blinded to the type of fluid being administered. Patients were randomized in the preoperative room prior to transfer to the operation theatre to either receive 0.9% normal saline (NS), Ringer’s lactate (RL) or Plasmalyte (PL).

Anaesthesia management

All the participants received premedication overnight and in the morning on the day of surgery with tab pantoprazole 40 mg, tab metoclopramide 10 mg and tab alprazolam 0.25 mg. Upon arrival to the operation theatre, ASA standard monitors like five-lead electrocardiography, heart rate, blood pressure and pulse oximetry were attached along with entropy and neuromuscular transmission (NMT) monitoring. Before induction of anaesthesia, an 18-G intravenous (IV) cannula was secured in fistula free arm. After preoxygenation, general anaesthesia was induced using IV propofol (2-2.5 mg/kg), fentanyl (2 µg/kg) and atracurium (0.5 mg/kg), and maintained with desflurane in an oxygen-nitrous oxide mixture at a 1:1 ratio. Desflurane was adjusted to maintain a state entropy of 40-50. Additional doses of atracurium were given as necessary to maintain a train-of-four (TOF) stimulation count of 1. A central venous catheter was inserted after induction of anaesthesia in the right internal jugular vein under ultrasound guidance for central venous pressure (CVP) monitoring and the radial artery was cannulated in fistula free arm for invasive arterial monitoring. The patient was kept on controlled mechanical ventilation to maintain end-tidal carbon dioxide tension near 35 mmHg. Tidal volume was set at 8 mL per kilogram lean body weight with positive end-expiratory pressure of 5 mmHg. Forced-air warming was used to keep patients normothermic. After completion of the surgery, the neuromuscular blockade was reversed and the patient was extubated and shifted to the post-renal transplant intensive care unit. Intraoperatively, all patients received 0.5-1 mg/kg frusemide as instructed by the surgical team.

Fluid management

IV crystalloids were given intraoperatively targeting pulse pressure variation (PPV) of 13% and a CVP of 12-14 mmHg as per our institute protocol, and the total volume of fluids administered in each group was recorded. After surgery, once urine output had been established, continuous infusion of IV fluids was administered targeting CVP of 14-16 mmHg in the post-renal transplant intensive care unit until discharge to the normal ward.

Management of intraoperative hypotension and dyselectrolytemia

Intravenous dopamine was administered when hypotension, defined as a mean arterial blood pressure less than 20% baseline, persisted after repeated fluid boluses totalling 10 ml/kg. Intraoperative hyperkalemia with signs of ECG changes (elevation in T-waves) was treated initially with calcium-gluconate 1 gm administered slowly over 10 minutes. In severe cases (i.e., presence of ECG changes and potassium ≥ 6 mmol/L), the dextrose-insulin infusion was administered.

Data and measurements

The following data were obtained from all the participants: age, sex, weight, height, total intraoperative fluid administered, total fluid administered until discharge from the post-operative intensive care unit, use of calcium-gluconate or dextrose-insulin infusions and use of inotropes.

Arterial blood gases (ABG) were obtained after induction of anaesthesia (T0), prior to clamping the iliac vein (T1), 10 minutes after reperfusion of the donated kidney (T2) and at the end of surgery (T3). The following variables were obtained by ABG analysis: pH, partial pressure of carbon dioxide (pCO2), bicarbonate, base excess, sodium, potassium, chloride and lactate.

Haemodynamics, renal condition, arterial blood gases, serum electrolytes and urine output were recorded intraoperatively and postoperatively. We gathered data on 24-hour urine output and serum creatinine on postoperative days one, two and seven. The follow-up period ended at this time point.

Outcome Measures

The primary objective was to detect if there was any difference in the in vivo pH at the end of surgery between the three types of fluids. The secondary outcome measures were (1) acid-base balance at four intraoperative time points (pH, pCO2, partial pressure of oxygen [pO2], bicarbonate [HCO3], base deficit, sodium, potassium and chloride were the values under consideration); and (2) serum creatinine and urine output on day one, two and seven of surgery as a measure of renal function.

Statistical analysis

An estimated sample size of 159 was calculated with the goal to demonstrate a moderate effect size of 0.25 (Cohen’s F, G*Power 3.1.9.4) with the pH difference at the end of surgery (T3) as the primary outcome measure. To cover for potential dropouts, we increased this sample size to 180. Continuous data have been represented as mean ± SD as they were normally distributed and analysis has been done using one-way ANOVA. Pairwise comparisons have been made post hoc where needed using Tukey honestly significant difference (HSD) for multiple comparisons. The final analysis has been done using SPSS (IBM Corp., released 2017, IBM SPSS Statistics for Windows, version 25.0, Armonk, NY).

## Results

**Figure 1:**
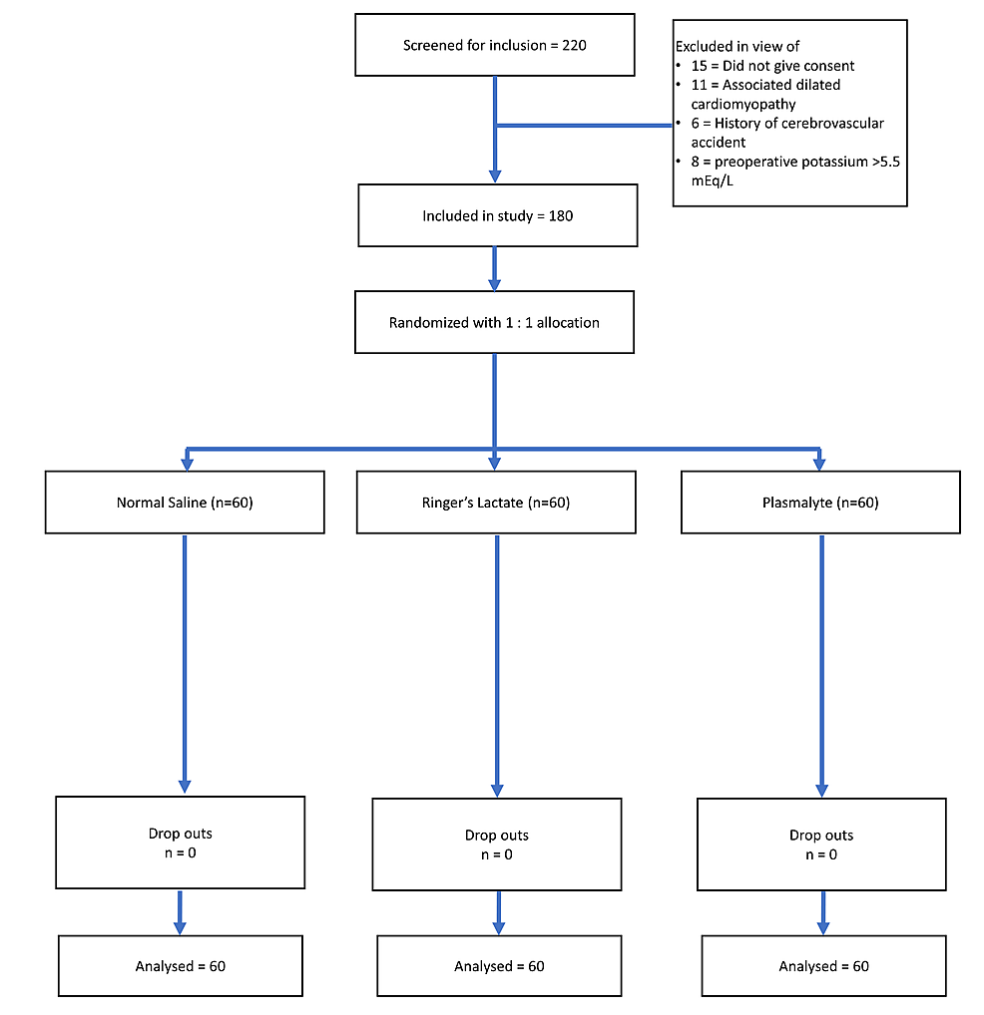
CONSORT flow diagram. CONSORT, Consolidated Standards of Reporting Trials.

A total of 220 patients were screened and 180 were randomized and included in the study between April 2015 and December 2018 (Figure 1). A total of 60 patients received normal saline (NS), 60 patients received Ringer’s lactate (RL) and 60 patients received acetate-buffered balanced crystalloid (Plasmalyte A, PL). All three groups were comparable in terms of age, sex, height and weight. Baseline patient characteristics and perioperative variables are listed in Table 1. There was no significant difference between groups with respect to donor cold ischemia time or duration of surgery (p > 0.05). During surgery, in order to maintain PPV of 13% and a CVP of 12-14 mmHg, patients in the NS group required a volume of 1895.00 ± 290.19 ml, which was higher than the volume needed in the other two groups (RL: 1497.50 ± 297.08 ml and PL: 1335.83 ± 189.13 ml, p < 0.001).

**Table 1:** Demographic and intraoperative parameters. Data are mean ± standard deviation unless otherwise stated. P-values represented are from one-way ANOVA comparing each parameter across the three fluid groups. * p < 0.05 is considered to be of statistical significance. # From Kruskal-Wallis test. NS = 0.9% normal saline group; RL = lactated Ringer’s solution group; PL = Plasmalyte group; N = 60 per group; IQR = interquartile range.

Parameter	NS	RL	PL	Sig
Age (years)	44.13 ± 12.47	45.15 ± 8.88	46.35 ± 15.64	0.631
Weight (kg)	59.18 ± 9.44	60.93 ± 6.40	59.06 ± 9.45	0.408
Height (cm)	165.33 ± 8.98	162.33 ± 5.96	164.90 ± 8.69	0.087
Charlson comorbidity index (median [IQR])	4 (4-5)	4 (3-5)	4 (3-4)	0.281#
Cold ischemia time (minutes)	30.73 ± 31.30	26.05 ± 3.89	28.20 ± 4.36	0.379
Anaesthesia time (minutes)	204.67 ± 17.47	203.32 ± 16.39	205.8 ± 17.31	0.713
Surgery time (minutes)	164.65 ± 20.39	166.62 ± 16.97	171.70 ± 17.69	0.099
Volume of fluid (ml)	1895 ± 290.19	1497.5 ± 297.08	1335.83 ± 189.13	<0.001*

The pH was balanced between groups at baseline (T0) (Table 2). Although clinically irrelevant, just before clamping the iliac vein (T1), those in the NS group had a lower mean pH 7.33 ±­ 0.05 while the highest pH was 7.38 ± 0.06 in the PL group (p = 0.001). On pairwise analysis, the pH differed significantly between NS and PL groups (mean difference [MD] −0.49, p = 0.003) but not between NS and RL (−0.004, p 0.947). At T2 (10 minutes after reperfusion), however, the pH was equally distributed between the groups. At T3 (end of surgery), the pH was lowest in the NS group at 7.29 ± 0.06 vs 7.33 ± 0.06 in RL group vs 7.31 ± 0.04 in the PL group (p = 0.03). Pairwise NS vs RL was not significantly different, while NS vs PL had a mean difference of −0.02 at a p-value of 0.024, RL vs PL had a mean difference of 0.02 at a p-value of 0.104. This shows that in terms of pH, balanced salt solutions perform better than NS. These changes were paralleled by changes in the serum chloride values along with changes in bicarbonate and base deficit. The difference in chloride was statistically significant (p < 0.001) at T2 with values qualifying for hyperchloremia (Cl > 110 mEq/L) being recorded in the NS group at 112.77 ± 5.12 mEq/L. This further increased to 118.49 ± 4.47 mEq/L at T3 in the NS group while the RL and PL groups had values <110 mEq/L (p < 0.001).

**Table 2:** Arterial blood gas parameters at various time points. Data are mean ± standard deviation unless otherwise stated. P-values represented are from one-way ANOVA comparing each parameter across the three fluid groups. * p < 0.05 is considered to be of statistical significance. NS = 0.9% normal saline group; RL = lactated Ringer’s solution group; PL = Plasmalyte group; N = 60 per group; T0 = after induction of anaesthesia; T1 = just before clamping of the iliac vein; T2 = 10 min after reperfusion of the donated kidney; T3 = at the end of surgery.

	T0	p-value	T1	p-value	T2	p-value	T3	p-value
pH								
NS	7.37 ± 0.09	0.327	7.33 ± 0.05	0.001*	7.27 ± 0.05	0.083	7.29 ± 0.06	0.03*
RL	7.36 ± 0.06		7.34 ± 0.06		7.29 ± 0.05		7.33 ± 0.06	
PL	7.35 ± 0.08		7.38 ± 0.06		7.30 ± 0.05		7.31 ± 0.04	
Bicarbonate mEq/L								
NS	21.01 ± 4.35	0.975	22.10 ± 1.88	0.053	19.36 ± 1.11	0.002*	21.08 ± 1.69	0.001*
RL	21.16 ± 3.36		22.14 ± 1.68		20.02 ± 1.34		22.4 ± 1.16	
PL	21.15 ± 4.65		23.08 ± 2.35		20.69 ± 2.03		22.01 ± 1.53	
Base excess/deficit mEq/L								
NS	−2.86 ± 3.5	0.116	−1.94 ± 1.90	0.56	−4.77 ± 1.19	0.001*	−2.98 ± 1.78	<0.001*
RL	−2.02 ± 4.31		−1.82 ± 1.69		−4.02 ± 1.30		−1.61 ± 1.19	
PL	−1.47 ± 3.09		−0.93 ± 2.32		−3.44 ± 1.95		−1.98 ± 1.52	
Lactate mmol/L								
NS	1.21 ± 0.12	0.818	1.45 ± 0.16	0.08	1.50 ± 0.20	0.371	1.35 ± 0.14	<0.001*
RL	1.22 ± 0.05		1.54 ± 0.20		1.56 ± 0.14		1.30 ± 0.10	
PL	1.21 ± 0.05		1.52 ± 0.19		1.54 ± 0.22		1.23 ± 0.03	
PO2 mmHg								
NS	194.94 ± 10.79	0.519	192.03 ± 10.99	0.001*	179.47 ± 11.54	0.208	173.74 ± 11.20	0.871
RL	192.28 ± 11.01		197.30 ± 12.58		183.73 ± 9.77		172.50 ± 21.71	
PL	192.28 ± 12.42		206.75 ± 17.97		183.50 ± 12.90		171.48 ± 20.44	
PCO2 mmHg								
NS	33.22 ± 2.86	0.245	35.25 ± 4.89	0.011	35.59 ± 5.6	0.088	41.37 ± 4.48	0.111
RL	38.49 ± 22.52		37.55 ± 4.28		35.45 ± 6.8		38.76 ± 6.78	
PL	36.12 ± 2.85		35.91 ± 3.46		33.42 ± 5.5		39.71 ± 4.43	
Chloride mEq/L								
NS	102.06 ± 3.23	0.129	111.70 ± 47.84	0.302	112.77 ± 5.12	<0.001*	118.49 ± 4.47	<0.001*
RL	103.13 ± 2.71		103.63 ± 4.03		103.55 ± 3.15		105.23 ± 1.85	
PL	101.83 ± 3.13		102.49 ± 4.51		103.93 ± 3.24		105.30 ± 1.96	
Potassium mEq/L								
NS	4.80 ± 0.91	0.214	4.80 ± 0.85	0.001*	5.12 ± 0.62	0.359	3.80 ± 0.54	<0.001*
RL	5.05 ± 0.71		5.60 ± 0.41		5.29 ± 0.42		3.45 ± 0.29	
PL	5.08 ± 0.54		5.29 ± 0.47		5.26 ± 0.54		4.04 ± 0.63	

**Figure 2:**
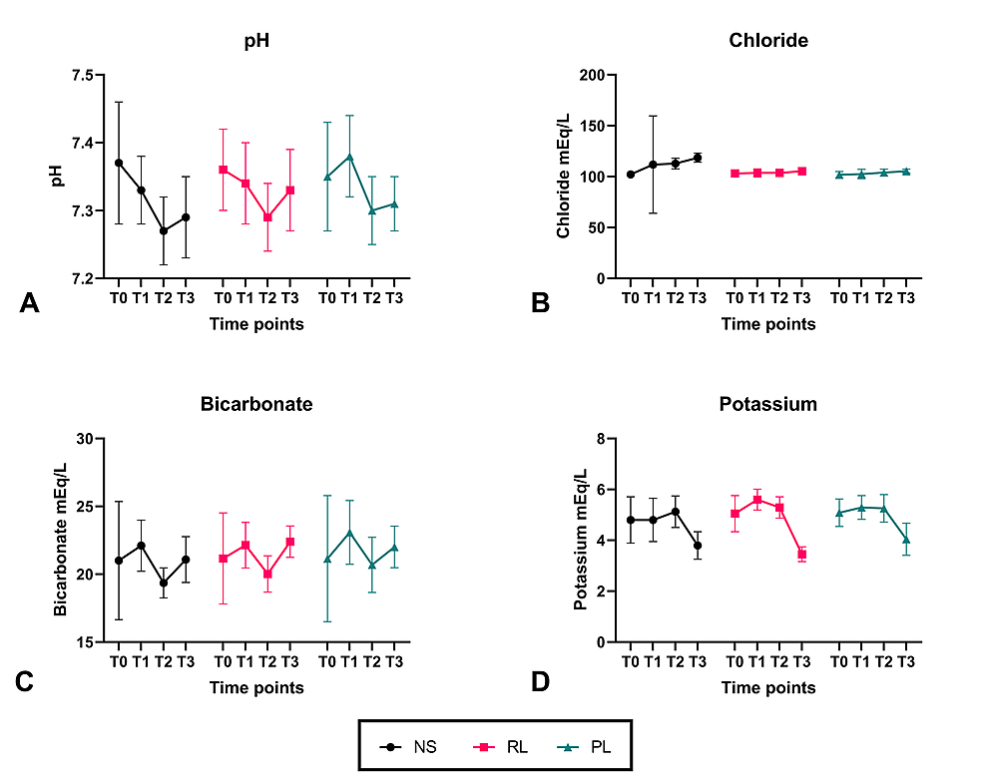
Progress of arterial blood gas parameters over different time points. NS = 0.9% normal saline group; RL = lactated Ringer’s solution group; PL = Plasmalyte group; N = 60 per group; T0 = after induction of anaesthesia; T1 = just before clamping of the iliac vein; T2 = 10 min after reperfusion of the donated kidney; T3 = at the end of surgery. The means of the respective variables have been plotted with the error bars representing SD (standard deviation).

Bicarbonate was also lowest in the NS group at T2 (19.36 ± 1.11), which differed significantly from the other two groups (p = 0.002; Figure 2). A similar trend was noted between the groups at T3. Lactate was statistically at its lowest in the PL group at T3, although the differences are not clinically relevant. Potassium was highest in the RL group (5.60 ± 0.41 mEq/L) at T1 and this resolved over time in all three groups at T2 and T3. This could be due to a return in renal function at this point. Although there was a statistically significant difference between the groups in potassium levels at T3, no patient was clinically hyperkalemic (defined as potassium > 5.5 mEq/L).

**Figure 3:**
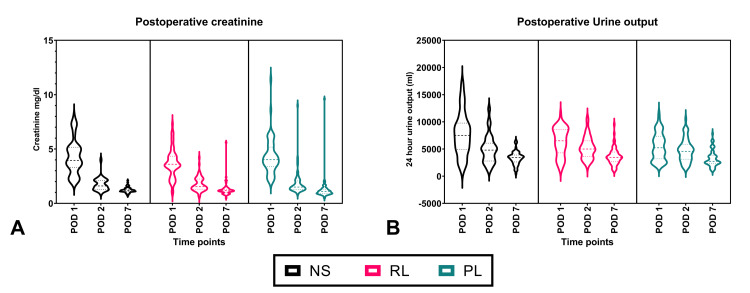
Violin plot of postoperative renal function parameters. The horizontal solid line represents the median while the horizontal discontinuous lines represent the interquartile range. NS = 0.9% normal saline group; RL = lactated Ringer’s solution group; PL = Plasmalyte group; N = 60 per group; POD = postoperative day.

There was no statistically significant difference in the creatinine values between the three groups from postoperative day one to day seven (Table 3 and Figure 3). However, urine output was highest in the NS group on day one (7744.42 ± 3926.63 ml/day) and lowest in the PL group (541.92 ± 2504.37 ml/day, p < 0.001). This trend did not carry forward into the second day and beyond where the urine output was equally distributed between the groups. None of the patients needed prolonged or high dose vasopressor use in the perioperative period. None of the recipients needed haemodialysis in the postoperative period till discharge.

**Table 3:** Postoperative renal function parameters. Values are represented as mean ± SD. P-values are from one-way ANOVA. * p < 0.05 is considered statistically significant. NS = 0.9% normal saline group; RL = lactated Ringer’s solution group; PL = Plasmalyte group; POD = postoperative day.

	Group	POD 1	p-value	POD 2	p-value	POD 7	p-value
Creatinine (mg/dl)	NS	4.26 ± 1.59	0.054	1.76 ± 0.61	0.821	1.22 ± 0.25	0.938
	RL	3.70 ± 1.33		1.76 ± 0.67		1.26 ± 0.66	
	PL	4.30 ± 1.64		1.85 ± 1.15		1.26 ± 1.14	
Urine output (ml/day)	NS	7744.42 ± 3926.63	<0.001*	5002.92 ± 2596.12	0.531	3323.08 ± 1208.04	0.178
	RL	6340.83 ± 2601.78		5226.50 ± 2051.32		4272.45 ± 5595.93	
	PL	5415.92 ± 2504.37		4749.67 ± 2085.55		3214.00 ± 1485.09	

## Discussion

The goal of our study was to see if the type of intraoperative fluid had any significant bearing on the acid-base status at the end of renal transplant surgery. Our findings suggest that there is statistically significant acidosis produced by normal saline when compared to balanced salt solutions. In our cohort, however, this did not translate to clinically significant acidosis (i.e., pH <7.25). The 95% CI for the pH in the NS group was 7.27-7.32, RL group was 7.31-7.36 and PL group was 7.29-7.33, p < 0.001. The mechanism of acidosis produced by NS has been attributed to hyperchloremia [1]. Even infusion of normal saline over a few hours has also been implicated in causing significant hyperchloremia and acidosis [4-8]. The suggested mechanisms by which hyperchloremia may cause acute kidney injury include a decreased glomerular filtration rate (GFR) due to increased chloride in the distal tubule, which in turn occurs due to reabsorption failure in the proximal tubule [9-11]. The other mechanisms include hyperchloremia-induced inflammatory mediator, thromboxane and cytokine release [12,13]. This acidosis and hyperchloremia have been known to cause renal vasoconstriction and reduced renal blood flow, which would be of great concern to a transplant recipient [14].

A 2015 systematic review, including 477 participants, on giving lower chloride solutions to renal transplant recipients also reported similar findings as our study [15]. These were that balanced salt solutions had a higher pH (MD of 0.07, 95% CI 0.05-0.09), higher serum bicarbonate and lower serum chloride (MD −9.93 mEq/L, 95% CI −19.96 to 0.11). However, none of the studies included reported significant adverse outcomes such as graft rejection or function that could be attributed to the type of fluids used. Similarly in our study, although participants in the NS group had a higher chloride and a lower pH, this did not translate into a persistently raised postoperative creatinine or poor postoperative urine output. None of the fluids produced clinically significant hyperkalemia suggesting that the balanced salt solutions are relatively safe to use perioperatively in transplant recipients under monitoring.

One of the limitations of our study includes the lack of a long-term follow-up in assessing graft survival. We did not prospectively assess 30-day mortality, although retrospectively all patients, in all groups, did arrive for scheduled follow-up visits even at 90 days post-transplant. This possibly means in our cohort the type of perioperative intravenous fluid did not significantly influence mortality. Assessing a biomarker such as neutrophil gelatinase-associated lipocalin (NGAL) or serum renalase either as part of the study or in the follow-up clinic may have given some additional information. We targeted CVP to ensure adequate preload as we were unable to convince the perioperative team on the robustness of PPV in the renal transplant population owing to poor evidence for the same and the prevailing institute management protocol at the time of study design.

## Conclusions

Balanced salt solutions such as Plasmalyte and Ringer’s lactate are associated with better pH and chloride levels compared to normal saline when used intraoperatively in renal transplant patients. The potassium kinetics also appear to be safe, further informing us that balanced salt solutions can be used safely during renal transplantation. This difference, however, does not appear to have any bearing on graft function. Plasmalyte seems to maintain a better acid-base and electrolyte balance, especially during the postreperfusion period.
